# Serous Pigment Epithelium Detachment Associated with Age-Related Macular Degeneration: A Possible Treatment Approach

**Published:** 2012

**Authors:** Nataliya V. Pasyechnikova, Volodymyr A. Naumenko, Andrii R. Korol, Oleg S. Zadorozhnyy, Taras B. Kustrin, Illya O. Nasinnyk

**Affiliations:** The Filatov Institute of Eye Diseases and Tissue Therapy of NAMS of Ukraine, Odessa, Ukraine

**Keywords:** Age-related macular degeneration, Serous pigment epithelium detachment, Triamcinolone acetonide

## Abstract

To evaluate the effects of intravitreal triamcinolone acetonide (TA) as a monotherapy of serous Pigment Epithelial Detachment (PED) associated with AMD (Age-Related Macular Degeneration), this study has been performed. Seventeen patients (19 eyes) with serous PED associated with AMD were observed. All patients received 0.1ml (4mg) of intravitreal TA. The mean follow-up period was 18 months. Re-attachment of serous PED was observed in 37% of cases to the end of follow-up. In other cases, the height and length of serous PED significantly decreased. Visual acuity remained stable in all cases. No evidence of RPE tear or CNV development were noted. Before TA administration, intraocular pressure (IOP) was 20.18 ± 2.58 mmHg however, after intravitreal TA, IOP increased gradually and reached its maximum of all period of observation (23.25±1.85mmHg) six months after injection (P=0.031). In 7 (37%) of the cases, progression to cataract was observed after treatment. After surgery, the visual acuity in all cases increased by 0.2 to 0.5. As a conclusion, intravitreal TA decreases of both the height and length of serous PED associated with AMD after 18 months follow-up in most cases. The presented data provides support for the hypothesis regarding the possibility of monotherapy of serous PED with intravitreal TA.

## INTRODUCTION

Serous retinal pigment epithelium detachment (PED) is one of the symptoms of age-related macular degeneration (AMD) and is detected in about 10% of patients with the exudative form of AMD [1-3]. Haab was the first researcher to clinically describe serous PED [2]. Later, through electron microscopy, it was confirmed that the detachment of RPE occurs between the inner layer of Bruch’s membrane and the basal membrane of RPE [4]. Its height and length fluctuates from tens of micrometres to several millimetres. PED is visualised in biomicro-ophthalmoscopy in the form of a dome-like formation passing into the vitreous with clear lines of yellow-gray colour. Shape may be various: round, oval, horseshoe-shaped. With the long-term existence of PED (1-3 years), visual acuity indicators may remain high [5]. In the natural course, PED may spontaneously disappear or increase, or even become complicated by RPE tears (in about 10% of cases) and the formation of choroidal neovascularition (CNV) in about 7% of cases [6-8].

## HYPOTHESIS

Several authors have proven the role of increased permeability of the vascular wall in the pathogenesis of the exudative form of AMD. Triamcinolone acetonide (TA) is a long-acting corticosteroid, which reduces vascular permeability. Therefore, the use of TA increases the potency of photodynamic therapy and anti-vascular endothelial growth factor therapy in wet AMD [9-11]. Given the avascular nature of serous PED, the presence of inflammation elements in pathogenesis, and the increased effectiveness of treating PED with a combination including TA, the hypothesis were suggested about using a monotherapy for this form of AMD with intravitreal TA.

## METHODS

Seventeen patients (19 eyes) with serous PED were included to be associated with AMD after obtaining ethical approval and informed consent. All of the patients received 0.1ml (4mg) of intravitreal TA. In the absence of positive dynamics of the structural parameters, re-injection was conducted 3 months after the previous administration of TA. As the means of control, data was used from the Moorfields Eye Hospital on 49 patients (49 eyes) which involved clinical research of the laser photocoagulation application in treatment of serous PED compared to its natural course [12]. Patients underwent visiometry, tonometry, fluorescein angiography, and optical coherence tomography (OCT). OCT examined the height and length of serous PED. Investigations was also carried out before the treatment and 1, 3, 6, 12, 15, 18 months after treatment. 

Statistical analysis performed with Statistica 8.0. The nonparametric Mann-Whitney test used to assess the statistical significance of differences between indicated groups, which determines the level of statistical significance.

## RESULTS

The average visual acuity of patients before treatment was 0.3. While undergoing OCT, the range of serous PED height was 115 to 1200 microns, and the length of the serous PED was 850 to 4660 microns. 

One month after TA administration, the visual acuity of patients increased to 0.35 (P = 0.43). The height of the serous PED decreased from 115-1200 microns to 82-1110 microns, and the length from 850-4660 microns to 610-3360 microns. Three months later, the average visual acuity was 0.3 (P = 0.27). The height of the serous PED was 82-1100 microns and the length of the serous PED was 540-3270 microns.

**Figure 1:**
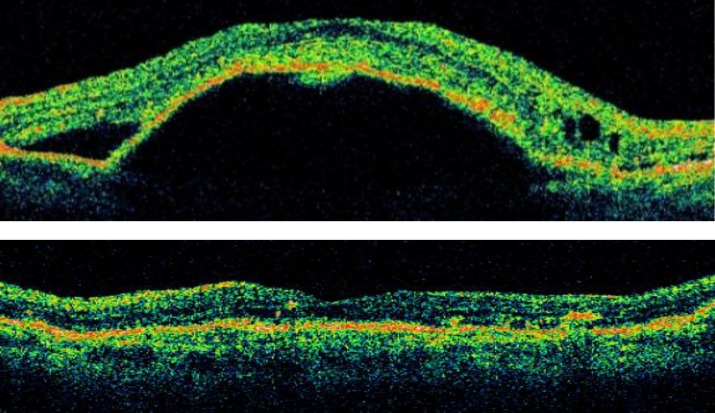
Serous PED before intravitreal TA (upper). Complete reattachment of PED after treatment in 6 month of follow-up (Lower).

On the sixth month of follow up, visual activity remained at the same level of 0.3 (P=0.16). The height of the serous PED decreased to 82-1050 microns, with the length of the serous PED being at the level of 628 to 3160 microns. Three patients (3 eyes) were observed to have complete reattachment of PED (Fig. 1).

**Figure 2:**
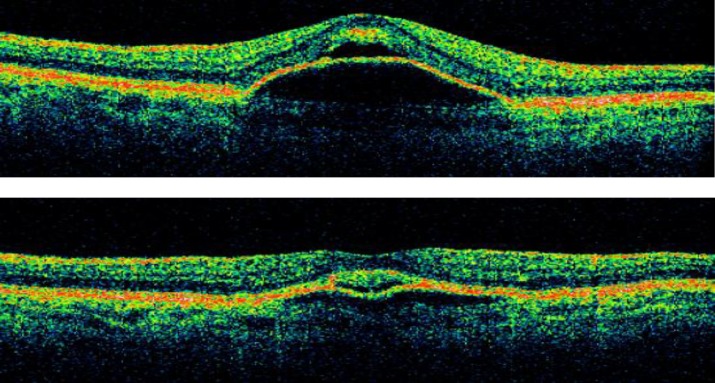
Serous PED before intravitreal TA (upper). Decreased height and length of serous PED after intravitreal TA in 18 months later (lower).

**Figure 3:**
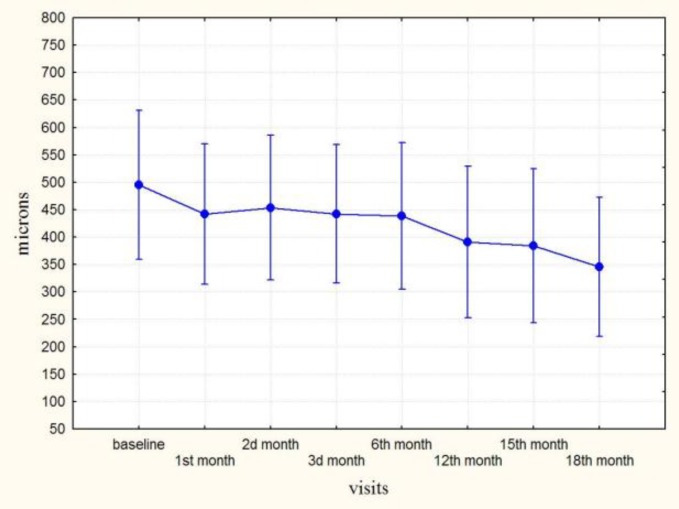
Height of serous PED after intravitreal TA in patients with persisting serous PED

Nine months later, visual acuity among the group of patients who had undergone TA administration was 0.29 (P=0.18). The height of serous PED ranged from 85 to 620 microns, and the length ranged from 280 to 3225 microns. The complete reattachment of serous PED was observed in 2 cases. Twelve months later, visual acuity remained unchanged, and was 0.29 (P = 0.15). The height of serous PED was 85-620 microns, and the length of serous PED was 280-3225 microns. One patient (1 eye) had complete reattachment of serous PED. Fifteen months later, the average visual acuity decreased to 0.27 (P = 0.13). The height of serous PED was 75-620 microns, and the length was 260-3225 microns. Complete reattachment of serous PED was observed in one case. By the 18^th^ month, visual acuity increased to 0.3 (P=0.1). The height of the serous PED was from 75-600 microns, and the length of serous PED was 260-3190 microns (Fig. 2, 3).

In all cases of complete reattachment, the initial structural characteristics were different. Thus, the height of serous PED ranged from 225 to 1200 microns, and the length was from 935 to 4660 microns. No connection between the size of serous PED, reattachment and the number of injections was reported. According to research carried out in the Moorfields Eye Hospital on 49 patients (49 eyes) whose visual acuity deteriorated, the size of RPE detachment preserved or increased over the 18 months of observation in the natural course of the disease [12].

**Figure 4:**
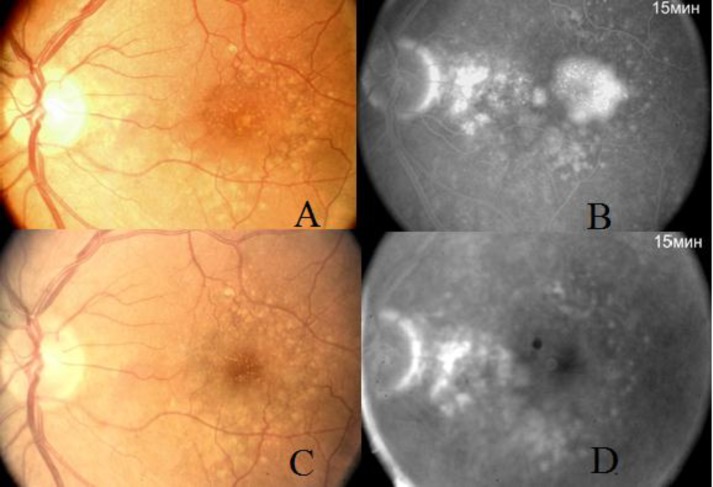
Color photo of fundus with serous PED before treatment (A). Angiography recirculation phase. Hyperfluorescence is observed in areas with serous PED (B). Colour photo of the same patient`s fundus after treatment (C). No hyperfluorescent area of PED in fovea has been noted (D).

Each patient underwent 1 or 2 intravitreal injections of TA. No RPE re-detachment was noted. During the whole observation period, complete reattachment of PED was observed in 7 out of 19 cases of intravitreal TA (Fig. 4). 

Prior to TA administration, the intraocular pressure (IOP) was 20.18 ± 2.58mmHg however, after intravitreal TA, the IOP rose gradually and reached its maximum observation of 23.25±1.85 mm Hg (P=0.031) six months after injection. In seven (37%) cases, progression of cataract was observed after treatment. In all cases, phacoemulsification with intraocular lens implantation was indicated. After surgery the visual acuity increased in all cases by 0.2-0.5.

## DISCUSSION

The management of PED encountered various controversies. Thus, with the intravitreal injection of anti-VEGF to the patients with serous PED, a tear of RPE was observed in 17% of patients one month after injection. The risk of RPE tear increased by 2-5 times after re-injection. As a result of 15-month long research following the photodynamic therapy carried out among patients with serous PED, an RPE tear was observed in 11% of cases [6,13,14].

Studies at the Moorfields Eye Hospital showed a reduction in visual acuity in 55% of cases (27 eyes) with the natural course of disease in the 9^th^ month of observation with a total observation period of 18 months. Visual acuity gradually decreased, and the size of RPE detachment either remained the same or increased. In 13% of cases an RPE tear had been observed. 45% of cases (22 eyes) with PED who underwent laser photocoagulation showed a decrease in visual acuity by the 3^rd^ month of observation. A decrease of PED was observed in most cases, however visual acuity continued to decline. A tear after laser treatment occurred in 10% of cases [12].

In this context, it became an issue to develop new effective approaches in the treatment of age-related macular degeneration with PED.

According to the literature, patients with the exudative form of AMD were found to have pro-inflammatory and angiogenic factors expressed by macrophages (TNF-α, IL-6, IL-10 and other cytokines), suggesting that increased vascular permeability plays a significant role in the pathogenesis of AMD [13,15]. Triamcinolone acetonide is a long-acting corticosteroid with a high anti-inflammatory effect which also reduces the permeability of the vascular wall. Also, a number of authors have shown that patients with wet AMD had improved results of CNV and PED treatment when using the combination of laser photocoagulation, photodynamic therapy, and anti-VEGF with TA [9,11]. Given the avascular nature of PED, the presence of inflammation elements in pathogenesis, and the increased effectiveness of treating PED with use of combination of TA, a hypothesis were suggested regarding the possibility of monotherapy in this form of AMD.

An intravitreal injection of triamcinolone acetonide provides a decrease of the height and length of serous PED for a period of 18 months, and also includes its complete reattachment in 37% of cases. As a result of treatment, visual acuity remained stable. There were no complications of serous PED associated with RPE tear or CNV development. The intravitreal injection of TA led to the progression of cataracts in 37% of cases, as well as a transient increase of IOP, which is consistent with published data [14-16].

## CONCLUSION

The presented data provides support for the hypothesis about the possibility of using monotherapy for serous PED with intravitreal TA. Thus, TA acts as anti-inflammation and non-direct anti-angiogenic factor and provides pathogenetic action on PED. Intravitreal injection of TA allows the complete reattachment of serous PED in 37% of cases, and the reduction of serous PED in 63% during the observation period of 18 months. Also, it allows the retention of visual acuity compared to the natural course and laser treatment in serous PED.

## DISCLOSURE

The authors report no conflicts of interest in this work.
